# Supplementary figures and images for: Dapagliflozin mitigates myocardial inflammation and metabolic stress in heart failure through STAT1 inhibition: Evidence from multi-omics analyses and experimental exploration
Source: PLoS One. 2026 Feb 27;21(2):e0343296. doi: 10.1371/journal.pone.0343296 (PMC12948057; doi:10.1371/journal.pone.0343296)

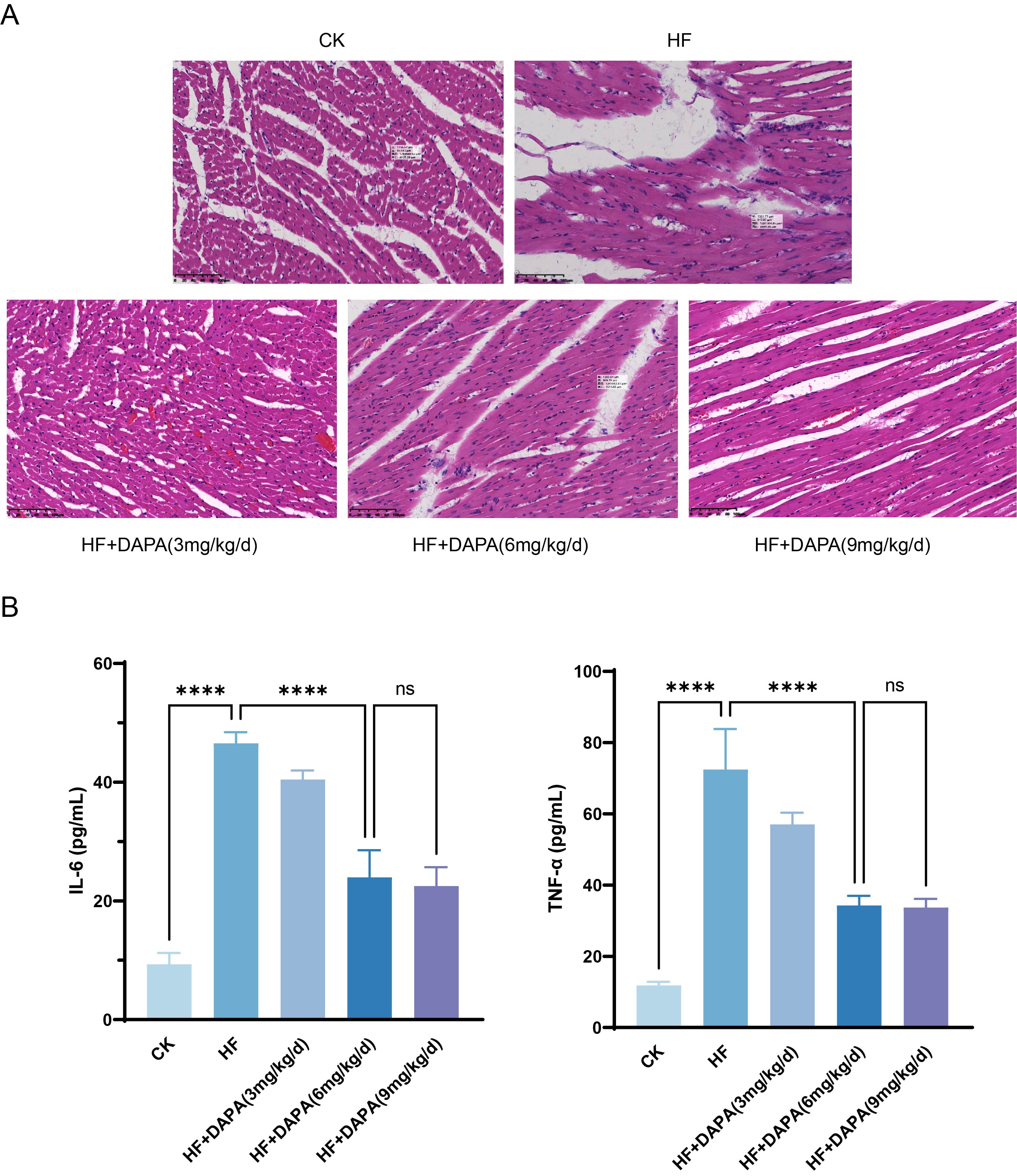

Supplement: S1 Fig — (A) and serum inflammatory cytokine levels (IL-6 and TNF-α) (B) in control (CK), heart failure (HF), and HF rats treated with dapagliflozin at 3, 6, or 9 mg/kg/day. (TIFF) [file pone.0343296.s001.tiff]

**STAT1**

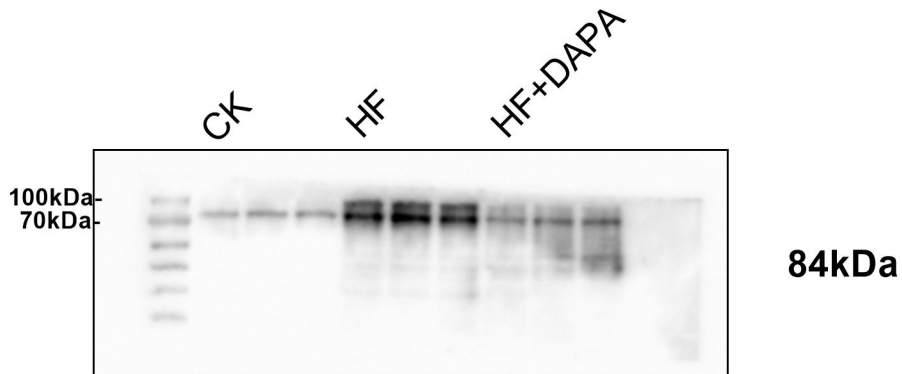

**GAPDH**

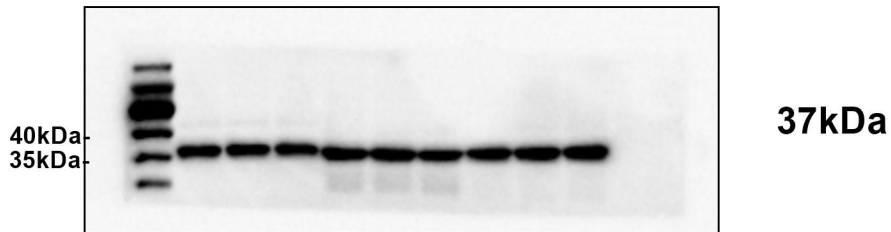

H9C2-NC  
H9C2-STAT1-ov

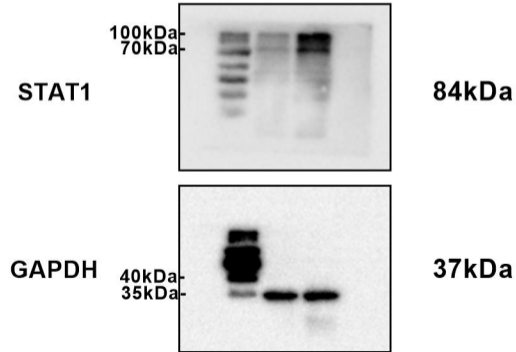

H9C2-NC  
H9C2-STAT1-ov

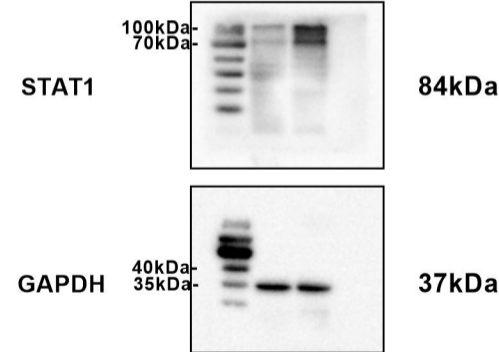

H9C2-NC  
H9C2-STAT1-ov

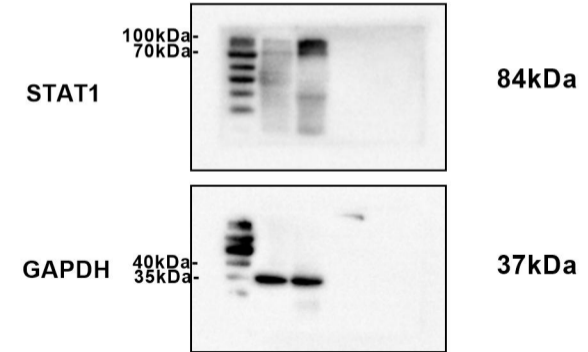

Supplement: S2 Fig — (PDF) [file pone.0343296.s002.pdf]
