# Supplementary material for: Dapagliflozin mitigates myocardial inflammation and metabolic stress in heart failure through STAT1 inhibition: Evidence from multi-omics analyses and experimental exploration
Source: PLoS One. 2026 Feb 27;21(2):e0343296. doi: 10.1371/journal.pone.0343296 (PMC12948057; doi:10.1371/journal.pone.0343296)
Supplement: Attachment — Submitted filename: Response to Reviewers(1).docx [file pone.0343296.s003.docx]

**Response to Reviewers**

Dear Editor and Reviewers,

We have revised the entire article, not only addressing the reviewer's comments but also reorganizing the logic of the text, reinterpreting important sections, and adding a detailed single-cell analysis section. This not only distinguishes and demonstrates the intrinsic differences between different cell types, but also emphasizes the differences in the STAT1 gene, which we focused on. Below is our point-by-point response.

**Response to Reviewer 1**

**1. G-418 concentration:**
The concentration is specified in Section 2.10 (Cell-based experiments) as 200 µg/mL for 48 hours for selecting stable STAT1-overexpressing H9C2 cells.

**2. Antibody concentrations:**
In Section 2.9 (Immunological assays), we have specified that cells were stained with APC-conjugated CD86 and PerCP/Cy5.5-conjugated CD163 antibodies for 30 min at 4°C. While specific dilutions were not provided in the original submission, we followed manufacturers' recommended protocols.

**3. Dataset designation:**
The correct dataset is GSE57345 throughout the manuscript. We have verified all references are consistent.

**4. Figure 1A visualization:**
We have revised Figure 1A to improve clarity with better color distinction between upregulated and downregulated genes and reduced label overlap.

**5. Formatting:**
Formatting issues have been corrected throughout.

**6. Log2FC threshold:**
In Section 2.1, we state the threshold as |log₂FoldChange| > 0.5 and P < 0.05 for identifying DEGs. This threshold was selected to balance statistical stringency with capturing biologically meaningful changes.

**7. DEG analysis strategy:**
We used all DEGs for comprehensive functional enrichment to capture the full landscape of transcriptional changes. Separate analyses of upregulated pathways are shown in Figure 3 for metabolic processes.

**8-10. Figure references:**
All figure references have been reviewed and corrected throughout the manuscript.

**Response to Reviewer 2**

**1. Statistical Rigor:**
Section 2.11 (Statistical Analysis and Visualization) describes our statistical approach:

- For single-cell analyses: Kruskal-Wallis test with Dunn's post hoc test and Benjamini-Hochberg correction
- For experimental validation: unpaired t-tests (two groups) or one-way ANOVA with Tukey's HSD (multiple groups)
- Sample sizes: n=6 per group for animal experiments, with each assay repeated at least three times for cell experiments
- All analyses performed in R environment with significance at P < 0.05

**2. Data Availability:**
As stated in our Data Availability section, we will supply relevant data in response to reasonable requests. The public datasets used (GSE57345 and GSE145154) are available from GEO.

**3. Experimental Design:**
We acknowledge that our study focuses on STAT1 as a key mediator but recognize DAPA likely has broader immunometabolic effects. Our in vitro experiments using STAT1-overexpressing cells (Section 3.11) specifically demonstrate DAPA's ability to suppress STAT1 and rescue cell viability.

**4. Interpretation of Results:**
We have been careful to frame our findings appropriately. The Discussion acknowledges that our data "suggest" and "indicate" relationships rather than claiming definitive causality.

**5. Figures and Quantification:**
Western blot quantification is provided in Figures 10B and 11D, with protein levels normalized to loading controls and statistical comparisons shown.

**6. Mechanistic Depth:**
Our STAT1 overexpression experiments (Figure 11) demonstrate that elevated STAT1 directly impairs cardiomyocyte viability and induces apoptosis, effects reversed by DAPA treatment, supporting a direct mechanistic link.

**Response to Reviewer 3**

**1. Novel contributions:**
Our study advances the field by: (1) first single-cell resolution mapping of STAT1 activity across cardiac cell types in HF; (2) demonstrating paradoxical STAT1 module elevation despite global JAK-STAT suppression (Figures 8-9); (3) establishing STAT1 inhibition as a mechanism of DAPA's cardioprotective effects.

**2. Molecular pathway details:**
Our analysis reveals STAT1 acts as a hub linking metabolic stress pathways (bile acid, amino acid metabolism) with immune dysregulation (M1/M2 imbalance). DAPA treatment suppresses STAT1 expression (Figure 10B), reduces inflammatory cytokines, and normalizes macrophage polarization.

**3. Dataset validation:**
While our primary analysis used GSE57345 for bulk RNA-seq, we validated immune findings using independent single-cell data (GSE145154) across multiple HF subtypes (DCM, ICM_MI, ICM_NMI), strengthening our conclusions.

**4. Model applicability:**
Our rat model represents ischemic HF, but our single-cell analysis included both ischemic (ICM) and non-ischemic (DCM) human samples, showing similar STAT1 alterations across HF etiologies.

**5. Clinical translation:**
This represents an important future direction. Our identification of STAT1 as a biomarker (Figure 5) provides foundation for future clinical studies correlating STAT1 levels with DAPA response in patients.

**6. Dose optimization:**
Figure S1A shows our pilot dosing study (3, 6, 9 mg/kg/day), identifying 6 mg/kg/day as most effective for reducing myocardial injury and inflammation.

**7. Inflammatory markers:**
IL-6 and TNF-α measurements are presented in Figure S1B, showing significant elevation in HF and marked reduction with DAPA treatment, corroborating our flow cytometry data on macrophage polarization.

**8. Clinical relevance and limitations:**
The Discussion acknowledges several limitations: retrospective dataset analysis, need for larger multi-omics validation, and requirement for prospective clinical studies. We frame STAT1 as a "promising" biomarker and "potential" therapeutic target, recognizing the translational steps still needed.

**Additional Improvements**

We have made several additional refinements:

- Clarified methodological details throughout
- Ensured consistent terminology and figure references
- Improved figure quality and labeling
- Enhanced discussion of study limitations and future directions

We believe these revisions have comprehensively addressed all reviewers' concerns and strengthened our manuscript.

Sincerely,
The Authors
