# Supplementary material for: Dapagliflozin mitigates myocardial inflammation and metabolic stress in heart failure through STAT1 inhibition: Evidence from multi-omics analyses and experimental exploration
Source: PLoS One. 2026 Feb 27;21(2):e0343296. doi: 10.1371/journal.pone.0343296 (PMC12948057; doi:10.1371/journal.pone.0343296)
Supplement: Attachment — Submitted filename: Response to Reviewers V2.docx [file pone.0343296.s004.docx]

**Journal Requirements:**

When submitting your revision, we need you to address these additional requirements.

1. Please ensure that your manuscript meets PLOS ONE's style requirements, including those for file naming.

We have carefully reviewed and ensured that our revised manuscript fully complies with PLOS ONE's style requirements.

2. To comply with PLOS One submissions requirements, in your Methods section, please provide additional information regarding the experiments involving animals and ensure you have included details on (1) methods of sacrifice, (2) methods of anesthesia and/or analgesia, and (3) efforts to alleviate suffering.

We thank for this important reminder. We have now expanded our Methods section to include comprehensive details on animal welfare and experimental procedures, like: "Specifically, rats were anesthetized with isoflurane and fixed in the supine position after hair removal. Electrocardiogram (ECG) was continuously monitored throughout the procedure. After sterile preparation, a thoracotomy was performed along the left sternal border at the 3rd–4th intercostal space using tissue scissors to expose the heart. The left atrial appendage was gently lifted with sterile toothed forceps to expose the aortic root. A surgical suture was passed around the proximal segment of the left anterior descending coronary artery near the superior margin of the heart, and three secure knots were tied to achieve complete ligation. The thoracic wall was then rapidly closed with sutures. Control rats underwent the same thoracotomy procedure without LAD ligation (sham operation). Post-operatively, all rats were placed in a warm recovery chamber and monitored until full consciousness was regained. Analgesics and antibiotics were administered as needed to minimize discomfort and prevent infection..."

3. Please note that PLOS One has specific guidelines on code sharing for submissions in which author-generated code underpins the findings in the manuscript. In these cases, we expect all author-generated code to be made available without restrictions upon publication of the work. Please review our guidelines at https://journals.plos.org/plosone/s/materials-and-software-sharing#loc-sharing-code and ensure that your code is shared in a way that follows best practice and facilitates reproducibility and reuse.

Regarding the code sharing requirements, we confirm that all author-generated code underpinning the findings in our manuscript has been made available without restrictions. We have uploaded all computational scripts and analytical code as supplementary files with our submission, including scripts for differential gene expression analysis, WGCNA analysis, machine learning models, and single-cell RNA-seq analysis.

4. We note that the grant information you provided in the ‘Funding Information’ and ‘Financial Disclosure’ sections do not match.
When you resubmit, please ensure that you provide the correct grant numbers for the awards you received for your study in the ‘Funding Information’ section.

We sincerely apologize for the discrepancy. We have now corrected and unified the grant information in both the 'Funding Information' and 'Financial Disclosure' sections to ensure complete consistency throughout the manuscript.

5. Thank you for stating the following financial disclosure:
“This study was supported by the talent Program of Fushun City of China (No. FSYC202107012).”

Please state what role the funders took in the study. If the funders had no role, please state: "The funders had no role in study design, data collection and analysis, decision to publish, or preparation of the manuscript."
If this statement is not correct you must amend it as needed.
Please include this amended Role of Funder statement in your cover letter; we will change the online submission form on your behalf.

We have amended our financial disclosure statement as requested. The complete and correct statement is: "This study was supported by the talent Program of Fushun City of China (No. FSYC202107012). The funders had no role in study design, data collection and analysis, decision to publish, or preparation of the manuscript." We have updated this statement in our cover letter accordingly.

6. We note that your Data Availability Statement is currently as follows: All relevant data are within the manuscript and its Supporting Information files.

Please confirm at this time whether or not your submission contains all raw data required to replicate the results of your study. Authors must share the “minimal data set” for their submission. PLOS defines the minimal data set to consist of the data required to replicate all study findings reported in the article, as well as related metadata and methods (https://journals.plos.org/plosone/s/data-availability#loc-minimal-data-set-definition).
For example, authors should submit the following data:
- The values behind the means, standard deviations and other measures reported;
- The values used to build graphs;
- The points extracted from images for analysis.
Authors do not need to submit their entire data set if only a portion of the data was used in the reported study.
If your submission does not contain these data, please either upload them as Supporting Information files or deposit them to a stable, public repository and provide us with the relevant URLs, DOIs, or accession numbers. For a list of recommended repositories, please see https://journals.plos.org/plosone/s/recommended-repositories.
If there are ethical or legal restrictions on sharing a de-identified data set, please explain them in detail (e.g., data contain potentially sensitive information, data are owned by a third-party organization, etc.) and who has imposed them (e.g., an ethics committee). Please also provide contact information for a data access committee, ethics committee, or other institutional body to which data requests may be sent. If data are owned by a third party, please indicate how others may request data access.

Regarding the Data Availability Statement, we would like to clarify that our study is based entirely on publicly available data from the GEO database. These datasets can be freely accessed by any researcher at <https://www.ncbi.nlm.nih.gov/geo/>. We have included complete information about these data sources, including their accession numbers and detailed descriptions, both in our manuscript's Methods section and in the supplementary files. Additionally, we have uploaded all analysis code to the Supporting Information files, which includes clear documentation of how to access and process these public datasets. The code files contain all necessary scripts to replicate our bioinformatics analyses, including the values behind means, standard deviations, and other measures reported, as well as the scripts used to generate all graphs and figures. Since all raw data are publicly accessible through GEO, and our analysis code is provided in the supplementary files, researchers have everything needed to fully replicate our study findings. Besides, we also have updated our Data Availability Statement in the manuscript to read: “All raw data are available from the corresponding author upon request.”

7. PLOS requires an ORCID iD for the corresponding author in Editorial Manager on papers submitted after December 6th, 2016. Please ensure that you have an ORCID iD and that it is validated in Editorial Manager. To do this, go to ‘Update my Information’ (in the upper left-hand corner of the main menu), and click on the Fetch/Validate link next to the ORCID field. This will take you to the ORCID site and allow you to create a new iD or authenticate a pre-existing iD in Editorial Manager.

We confirm that the corresponding author's ORCID iD has been validated in the Editorial Manager system as required. The ORCID iD is now properly linked and authenticated in our submission.

8. PLOS ONE now requires that authors provide the original uncropped and unadjusted images underlying all blot or gel results reported in a submission’s figures or Supporting Information files. This policy and the journal’s other requirements for blot/gel reporting and figure preparation are described in detail at https://journals.plos.org/plosone/s/figures#loc-blot-and-gel-reporting-requirements and https://journals.plos.org/plosone/s/figures#loc-preparing-figures-from-image-files. When you submit your revised manuscript, please ensure that your figures adhere fully to these guidelines and provide the original underlying images for all blot or gel data reported in your submission. See the following link for instructions on providing the original image data: https://journals.plos.org/plosone/s/figures#loc-original-images-for-blots-and-gels.

In your cover letter, please note whether your blot/gel image data are in Supporting Information or posted at a public data repository, provide the repository URL if relevant, and provide specific details as to which raw blot/gel images, if any, are not available. Email us at plosone@plos.org if you have any questions.

We thank the editor for this important reminder regarding blot/gel reporting requirements. We have now prepared and uploaded the original, uncropped, and minimally adjusted images for all Western blot results reported in our manuscript. All original blot images are now available in the Supporting Information file S1_raw_images.pdf. And we have added the description to the cover letter.

9. We notice that your supplementary figures are uploaded with the file type 'Figure'. Please amend the file type to 'Supporting Information'. Please ensure that each Supporting Information file has a legend listed in the manuscript after the references list.

We have now amended the file type of all supplementary figures from 'Figure' to 'Supporting Information' in the submission system. Additionally, we have ensured that each Supporting Information file has a corresponding legend listed in the manuscript after the references list, as required by PLOS ONE guidelines.

10. If the reviewer comments include a recommendation to cite specific previously published works, please review and evaluate these publications to determine whether they are relevant and should be cited. There is no requirement to cite these works unless the editor has indicated otherwise.

We have carefully reviewed all reviewer comments and confirm that no specific previously published works were recommended for citation by the reviewers. Therefore, no additional citations based on reviewer recommendations have been added to the revised manuscript.

**Reviewer #1:** In the present study authors performed comptehensive bioinformatic and experimental analysis of the role of STAT1 in the heart failure and the effect of dapagliflozin. The results show that heart failure induced by coronary artery ligation is associated with the up-regulation of myocardial STAT1 whereas dapagliflozin reduced its expression. Dapagliflozin reduced the abundance of circulating M1 and increased M2 macrophages in rats with heart failure. DAPA lso reduced the level of inflammatory cytokines and total bile acids. In vitro, STAT1 overexpression reduced viability of H9C2 cardiomyocytes; the effect suppressed by dapagliflozin.
The topic and the results are of interest, however, there are also some important concerns to be addressed.

Dear Reviewer #1,

We sincerely thank you for your thorough and insightful review of our manuscript. Your comprehensive evaluation and constructive suggestions have substantially improved the quality and rigor of our work. We are particularly grateful for your attention to methodological details, statistical considerations, and interpretative nuances that we had not adequately addressed in the original submission.

Your thoughtful comments regarding the generalizability of our findings to the SGLT2 inhibitor class, the importance of sex differences in heart failure research, and the distinction between biomarker identification and mechanistic validation have prompted us to think more critically about the scope and limitations of our study. These insights have not only strengthened our current manuscript but have also deepened our understanding of the research questions and guided our thinking about future experimental directions.

We have carefully addressed each of your comments point-by-point and made corresponding revisions throughout the manuscript. The detailed responses to your specific comments are provided below. We believe these revisions have significantly enhanced the scientific rigor, transparency, and interpretative accuracy of our work.

Once again, we greatly appreciate the time and expertise you devoted to reviewing our manuscript. Your rigorous evaluation has been invaluable in refining our research.

1. Only one SGLT inhibitor was used in the experiments. Could the results be extrapolated to the whole class of drugs or apply only do dapagliflozin?

Thank you for raising this insightful question regarding the generalizability of our findings. We agree that since only dapagliflozin (DAPA) was used in our experimental setup, the direct conclusion that DAPA mitigates heart failure through STAT1 inhibition strictly applies to this specific agent.

However, based on extensive clinical evidence and proposed mechanistic overlap, we believe there is a strong rationale for a potential class effect concerning this pathway. The SGLT2 inhibitor class (including DAPA and Empagliflozin) has consistently demonstrated a class effect in major clinical trials (such as DAPA-HF and EMPEROR trials), significantly reducing heart failure hospitalization and cardiovascular death across different patient populations, irrespective of diabetes status. This suggests that the cardioprotective benefits are driven by mechanisms common to the class. Given that STAT1 is a crucial mediator of inflammation and metabolic stress—both universal pathological features of heart failure targeted by SGLT2 inhibitors—we hypothesize that STAT1 suppression represents a shared, pleiotropic, downstream target for the entire SGLT2 inhibitor class.

Nonetheless, we acknowledge this as a limitation of our current study, and we have added a statement to the Discussion section to address this point and suggest future comparative studies to definitively confirm the proposed class effect: “The robust evidence of a class effect for SGLT2 inhibitors in reducing HF hospitalization and cardiovascular mortality[37, 38] suggests that the fundamental mechanisms, such as STAT1 inhibition and subsequent immune/metabolic modulation, may be shared across the class. Future studies directly comparing the effects of different SGLT2 inhibitors (e.g., Dapagliflozin vs. Empagliflozin) on the STAT1 pathway are warranted to definitively confirm this proposed class effect.”

2. The method of LAD ligation including anesthesia and recovery should be described in more details.

We thank the reviewer for this important suggestion. We have now expanded the Methods section to provide more detailed descriptions of the LAD ligation procedure, including anesthesia protocols and post-operative recovery procedures:

"Myocardial infarction–induced heart failure (HF) was created in the experimental group by ligation of the left anterior descending (LAD) coronary artery. Specifically, rats were anesthetized with isoflurane and fixed in the supine position after hair removal. Electrocardiogram (ECG) was continuously monitored throughout the procedure. After sterile preparation, a thoracotomy was performed along the left sternal border at the 3rd–4th intercostal space using tissue scissors to expose the heart. The left atrial appendage was gently lifted with sterile toothed forceps to expose the aortic root. A surgical suture was passed around the proximal segment of the left anterior descending coronary artery near the superior margin of the heart, and three secure knots were tied to achieve complete ligation. The thoracic wall was then rapidly closed with sutures. Control rats underwent the same thoracotomy procedure without LAD ligation (sham operation). Post-operatively, all rats were placed in a warm recovery chamber and monitored until full consciousness was regained. Analgesics and antibiotics were administered as needed to minimize discomfort and prevent infection."

3. Sex of the animals used in the experiments should be specified. If only one sex was used, it should be discussed whether the results could be extrapolated to the other one.

We appreciate the reviewer's important comment regarding the sex of experimental animals. This study exclusively used male rats for several methodological reasons. First, the establishment of heart failure models, particularly through surgical approaches such as coronary artery ligation or aortic constriction, may be influenced by the estrous cycle in female rats. The cyclical fluctuations in estrogen and progesterone levels—both hormones known to exert cardioprotective and vasoactive effects—could introduce significant variability in infarct size, cardiac remodeling progression, and therapeutic responsiveness during the critical early phases of model establishment. The use of male animals was intended to minimize hormonal confounding factors. Second, as a fundamental mechanistic study, the use of male animals allows more direct comparison with existing literature, thereby facilitating initial validation of the proposed mechanisms.

However, we acknowledge important limitations regarding the extrapolation of our findings to female subjects. The pathophysiology of heart failure exhibits well-documented sex differences, including distinct patterns of hypertrophy, fibrosis, ionic remodeling, and drug metabolism. Therefore, the results of this study, which are derived entirely from male animals, should not be directly extrapolated to female individuals. The therapeutic effects and underlying mechanisms observed in our study require explicit validation in future investigations employing female animals or sex-balanced experimental designs. Understanding sex-specific responses to DAPA and STAT1-mediated pathways represents an important direction for translational research in heart failure management.

We have enriched the discussion: “Additionally, this study exclusively used male rats to minimize hormonal variability associated with the estrous cycle, which could confound infarct size and cardiac remodeling during model establishment. However, given well-documented sex differences in heart failure pathophysiology—including patterns of hypertrophy, fibrosis, and drug metabolism—our findings should not be directly extrapolated to female subjects and require validation in sex-balanced experimental designs.”

4. The vehicle used to dissolve dapagliflozin should be specified. Were animals not treated with dapagliflozin receiving any vehicle?

We thank the reviewer for this important methodological clarification. We have now specified the vehicle used to dissolve dapagliflozin and clarified that control animals received the same vehicle:

"Dapagliflozin was dissolved in 0.5% carboxymethylcellulose sodium (CMC-Na; HY-Y0703, MedChemExpress, USA) as the vehicle. Accordingly, from the second day after surgery, the HF + DAPA group was treated daily with oral gavage of dapagliflozin (HY-10450; MedChemExpress, USA, 6 mg/kg/day) dissolved in 0.5% CMC-Na for four weeks, while the HF group received an equivalent volume of 0.5% CMC-Na vehicle alone. The control group (CK) also received 0.5% CMC-Na vehicle."

5. Lines 194/195, the method of animal anesthesia and sacrifice for final tissue sampling should be described.

We thank the reviewer for this suggestion. We have enriched the description: “Myocardial infarction–induced heart failure (HF) was created in the experimental group by ligation of the left anterior descending (LAD) coronary artery. Specifically, rats were anesthetized with isoflurane and fixed in the supine position after hair removal. Electrocardiogram (ECG) was continuously monitored throughout the procedure. After sterile preparation, a thoracotomy was performed along the left sternal border at the 3rd–4th intercostal space using tissue scissors to expose the heart. The left atrial appendage was gently lifted with sterile toothed forceps to expose the aortic root. A surgical suture was passed around the proximal segment of the left anterior descending coronary artery near the superior margin of the heart, and three secure knots were tied to achieve complete ligation. The thoracic wall was then rapidly closed with sutures. Control rats underwent the same thoracotomy procedure without LAD ligation (sham operation). Post-operatively, all rats were placed in a warm recovery chamber and monitored until full consciousness was regained. Analgesics and antibiotics were administered as needed to minimize discomfort and prevent infection... Before tissue sampling, deep anesthesia was administered via intraperitoneal injection of sodium pentobarbital (200 mg/kg) until respiratory arrest. Blood samples were collected from the orbital venous plexus, and hearts were harvested at sacrifice four weeks post-modeling.”

6. Section 2.8, which part of the heart was used for the analysis (infarcion area or non-infarcted area?)

We thank the reviewer for seeking this clarification. We have now specified that the infarction area was used for all histological and protein expression analyses:

"For histopathology, myocardial tissues from the infarction area were fixed in 4% paraformaldehyde, dehydrated in graded sucrose, embedded in paraffin, and sectioned."

"For protein expression, heart tissues from the infarction area were minced and homogenized in cold lysis buffer containing protease and phosphatase inhibitors..."

7. The names, catalogue numbers and dilution rates of primary and secondary antibodies used for Western blotting should be specified.

We thank the reviewer for this important methodological detail. We have now provided complete information regarding all antibodies used for Western blotting, including their names, catalogue numbers, sources, and dilution rates:

"After blocking with 5% BSA for 1 h, membranes were incubated overnight at 4 °C with primary antibodies against STAT1 (1:1000 dilution; ET1612-22, Huabio, China) or GAPDH (1:5000 dilution; AF7021, Affinity, China) as the loading control. After washing three times with TBST, membranes were probed with goat anti-rabbit IgG secondary antibody (1:50,000 dilution; HA1001, Huabio, China) for 1 h at room temperature, and visualized using ECL substrate (AIWB-006; Affinity-life, China)."

8. Section 2.9, limits of quantification as well as intra- and inter-assay CV values for ELISA kits should be presented.

We thank the reviewer for requesting this important analytical information. We have now included the detection ranges, sensitivity, and intra- and inter-assay coefficient of variation (CV) values for all ELISA kits used in the study:

"Serum inflammatory cytokines were quantified using ELISA kits with absorbance recorded at 450 nm. TNF-α was measured using a rat TNF-α ELISA kit (CSB-E11987r-IS, Cusabio, China) with a detection range of 15.6–1000 pg/ml, sensitivity of 3.9 pg/ml, intra-assay CV <8%, and inter-assay CV <10%. IL-6 was measured using a rat IL-6 ELISA kit (CSB-E04640r, Cusabio, China) with a detection range of 0.312–20 pg/ml, sensitivity of 0.078 pg/ml, intra-assay CV <8%, and inter-assay CV <10%. Concentrations were calculated by four-parameter logistic regression. Total bile acid (TBA) levels in serum were measured using a colorimetric assay kit (E003-2-1; Nanjing Jiancheng Institute, China) with a detection range of 0–180 μmol/L, intra-assay CV ≤5.0%, and inter-assay CV ≤10.0%, according to the manufacturer's protocol."

9. The principle of the assay of bile acid concentration should be described.

We thank the reviewer for this request. We have now included a description of the enzymatic principle underlying the bile acid concentration assay:

"Total bile acid (TBA) levels in serum were measured using a colorimetric assay kit (E003-2-1; Nanjing Jiancheng Institute, China) with a detection range of 0–180 μmol/L, intra-assay CV ≤5.0%, and inter-assay CV ≤10.0%. The assay is based on enzymatic cycling reactions: bile acids are oxidized by 3α-hydroxysteroid dehydrogenase (3α-HSD) in the presence of S-NAD to produce 3-keto steroids and S-NADH; subsequently, 3-keto steroids are reduced back by S-NADH through diaphorase to regenerate NAD⁺ and bile acids. This cycling amplification reaction produces a color change at 405 nm, with the absorbance proportional to the bile acid concentration in the sample[35]."

10. Line 262, what does it mean that each assay was performed at least three times? Were, for example, cytokines measured in three samples from each animals?

We thank the reviewer for seeking this important clarification regarding our experimental replicates. We have now specified the distinction between biological and technical replicates for each assay type:

"For in vivo experiments, Western blotting, histological analyses (H&E staining), ELISA assays, and flow cytometry were performed using three randomly selected animals per group (n=3 biological replicates). For ELISA assays specifically, each biological sample was measured in duplicate (technical replicates). For in vitro experiments, Western blotting and qPCR analyses were performed with three independent cell culture experiments (n=3 biological replicates), while CCK-8 assays were performed with technical replicates within each experiment."

11. Statistical analysis, was normality of data distribution verified to justify using parametric tests? If so, what method was used?

We thank the reviewer for this important statistical clarification. Yes, normality of data distribution was verified prior to applying parametric tests. We have now included this information in the statistical analysis section:” In the experimental validation section, normality of data distribution was verified using the Shapiro-Wilk test prior to parametric analysis. All datasets met the assumption of normal distribution…”

12. Line 325, that STAT1 is the top contributor for distinguishing HF from control samples indicates only that its expression changes to the greater extent but not that it is the hub molecule in HF pathogenesis.

Thank you for this important clarification. We agree that ranking as the top contributor in random forest analysis reflects the magnitude of expression change rather than directly establishing STAT1 as a hub molecule in HF pathogenesis. We have revised the text to distinguish between STAT1's discriminative power as a biomarker (based on expression differences) and its role as a hub regulator (supported by WGCNA network topology and functional evidence). The revised statement now clarifies that STAT1's top ranking indicates pronounced expression change, while its hub molecule status is supported by its central position in the co-expression network and its known regulatory functions in inflammatory and metabolic pathways：“Combined with its central position in the WGCNA co-expression network and its known role in coordinating inflammatory and metabolic pathways, these findings suggest that STAT1 serves not only as a robust potential biomarker for HF classification but also as a key regulatory molecule warranting further mechanistic investigation in HF pathogenesis.”

13. Line 424, where is the evidence that DAPA reduced fibrosis? How do you conclude about correction of metabolic abnormalities?

Thank you for this critical observation. We acknowledge that our original statements overstated the evidence regarding fibrosis and metabolic abnormalities. Upon careful review, we recognize that: (1) While H&E staining showed inflammatory infiltration and tissue disorganization in HF hearts that were improved by DAPA, we did not perform specific fibrosis staining to directly quantify collagen deposition. Therefore, our conclusion about "reduced fibrosis" was not adequately supported. (2) Regarding metabolic abnormalities, our measurement was limited to serum total bile acid levels. While TBA reduction suggests improvement in one aspect of metabolic dysregulation identified in our bioinformatics analysis, this single parameter is insufficient to claim comprehensive "correction of metabolic disturbances."

We have revised Section 3.10 to accurately reflect the scope of our experimental evidence, focusing on the demonstrated anti-inflammatory effects, improved tissue architecture, normalization of macrophage polarization, reduced cytokine levels, and decreased bile acid levels, while removing overstated claims about fibrosis and broad metabolic correction.

14. Was the effect of STAT1 overexpression on H9C2 cells examined at physiological conditions or under oxygen deprivation?

We thank the reviewer for seeking this clarification. The effect of STAT1 overexpression on H9C2 cells was examined under standard normoxic cell culture conditions (physiological conditions). Cells were not subjected to oxygen deprivation or hypoxic treatment during these experiments. We have clarified in the manuscript:“All cell culture experiments were conducted under standard normoxic conditions (37°C, 5% CO₂, atmospheric oxygen levels ~21% O₂) without oxygen deprivation or hypoxic treatment.”

15. What is the evidence that STAT1 was involved in the effect of DAPA? Could down-regulation of STAT1 be the consequence rather than the underlying mechanism of its protective effect?

We sincerely thank the reviewer for raising this critical question regarding the causal relationship between STAT1 and DAPA's cardioprotective effects. This is indeed an important consideration that deserves careful discussion.

Evidence supporting STAT1 involvement in DAPA's effects:

1. Multi-level convergence of STAT1 as a key regulator: Our bioinformatics analyses (WGCNA, random forest classification, and hub gene identification) consistently identified STAT1 as a central node linking metabolic stress and immune dysregulation in heart failure, independent of any DAPA treatment.

2.In vivo intervention studies: DAPA treatment significantly reduced myocardial STAT1 expression in HF rats (Figure 10B), which was accompanied by coordinated improvement of STAT1-related pathological features including macrophage polarization, inflammatory cytokine levels, bile acid metabolism, and myocardial tissue architecture.

3.In vitro gain-of-function experiments: STAT1 overexpression alone was sufficient to induce cardiomyocyte injury. DAPA treatment suppressed STAT1 expression in STAT1-overexpressing cells and rescued the injury phenotype, demonstrating that DAPA can directly counteract STAT1-mediated cellular injury.

However, we acknowledge that our current study does not definitively exclude the possibility that STAT1 downregulation could be, at least in part, a consequence of DAPA's protective effects through other primary mechanisms (e.g., metabolic improvement or oxidative stress reduction). The relationship between STAT1 and DAPA's cardioprotection may be bidirectional or part of a complex regulatory network.

To more definitively establish causality, future experiments including STAT1 loss-of-function studies (knockdown/knockout followed by DAPA treatment), temporal relationship studies, and rescue experiments with constitutively active STAT1 would be valuable to determine whether STAT1 suppression is necessary and sufficient for DAPA's protective effects.

**Reviewer #2:** I understand that this is the second version of the manuscript. I did not review the first version therefore my comments are based solely on the second version. The following points need to be addressed:

Dear Reviewer #2,

We are deeply grateful for your careful and critical review of our manuscript. Your insightful comments have identified several important issues that required correction and clarification, particularly regarding data sources, experimental evidence, and the precision of our scientific claims.

We especially appreciate your rigorous scrutiny of our claims regarding fibrosis and STAT1 activity versus expression. These critical observations have prevented potentially misleading statements in our manuscript and have prompted us to re-examine our data interpretation with greater care and precision. Your expertise has been instrumental in ensuring that our conclusions accurately reflect the experimental evidence we present.

Your comments regarding the clarity of data sources (publicly available datasets versus our own experimental work) and the proper completion of ethics and data availability statements have also improved the transparency and reproducibility of our research reporting.

We have thoroughly addressed each of your concerns with detailed point-by-point responses below and have made extensive revisions throughout the manuscript, including systematic correction of terminology (expression vs. activation), removal of unsupported claims, explicit clarification of data sources, and addition of appropriate limitations in the Discussion section.

We believe your thoughtful and rigorous review has significantly elevated the scientific quality and accuracy of our manuscript. Thank you for your invaluable contribution to improving our work and for helping us communicate our findings more precisely and responsibly.

1) In the submission form, the authors inserted "N/A" in Ethics Statement section. Since they have undertaken animal studies, this section needs to be completed accordingly.

We apologize for this oversight. We have now completed the Ethics Statement section in the submission form.

2) In the same form in Data availability section and the authors stated "Yes - all data are fully available without restriction" and "All relevant data are within the manuscript and its Supporting Information files" which are not entirely correct. If possible the authors should state "all raw data are available from the corresponding author upon request". If that is not possible, it should also be stated. This needs to be mentioned also in the manuscript.

Thank you for your comment. Regarding the data availability statement, we have revised it to accurately reflect the accessibility of our data. We have updated both the Editorial Manager submission form and the Data Availability section in our manuscript to state: "All raw data are available from the corresponding author upon request." Additionally, we have provided detailed information about the publicly available datasets obtained from GEO database with their accession numbers clearly stated in the manuscript. All analysis code necessary to reproduce our results has been uploaded as supplementary files. This revised statement now accurately represents the availability of all data and materials used in our study.

3) Lines 55-57 is a very poor description of HF, please rephrase.

We sincerely thank the reviewer for this constructive feedback. We agree that our original description of heart failure was poorly constructed and did not accurately reflect the current understanding of HF as a clinical syndrome. We have revised this sentence to provide a more accurate and clinically appropriate definition：“Heart failure (HF) is a complex clinical syndrome characterized by the inability of the heart to pump sufficient blood to meet the body's metabolic demands, resulting from structural or functional cardiac abnormalities that impair ventricular filling or ejection [1].”

4) Lines 127-140: Human samples are mentioned here. Is this from available data set or did the authors analyse human cells? Please clarify.

We sincerely thank the reviewer for seeking this clarification. We apologize for the confusion caused by our imprecise wording. We did not analyze human cells ourselves. All single-cell RNA sequencing data were obtained from the publicly available GSE145154 dataset, which contains human cardiac tissue samples. We performed bioinformatic re-analysis of this existing dataset. To clarify this important point, we have revised Section 2.3 as follows:"For the publicly available human single-cell RNA sequencing data obtained from the GSE145154 dataset, quality control (QC) metrics were computed at the single-cell level, including mitochondrial transcript proportion, ribosomal gene proportion, and hemoglobin gene proportion..."

5) Lines 341-368: "single-cell transcriptomic analysis of myocardial tissues" was this done in available data set or did the authors gather this data from tissues they had? Please clarify.

We sincerely thank the reviewer for identifying this critical ambiguity in our Results section. We apologize for the misleading wording. We did not generate single-cell transcriptomic data from tissues ourselves. All single-cell data were obtained from the publicly available GSE145154 dataset and re-analyzed bioinformatically.

The phrase "we performed a systematic single-cell transcriptomic analysis" refers to our computational re-analysis of existing public data, not laboratory generation of new single-cell sequencing data.

We have revised Section 3.7 to eliminate this confusion: "To further validate the immune infiltration analysis and characterize the immune microenvironment of failing hearts under metabolic stress, we performed computational re-analysis of publicly available single-cell transcriptomic data from human myocardial tissues (GSE145154 dataset) ..."

6) Lines 370-386: "we calculated enrichment scores for single-cell data" which dataset was this? Please clarify.

We sincerely thank the reviewer for requesting this clarification. We apologize that the dataset source was not explicitly stated. The enrichment scores were calculated from the publicly available GSE145154 single-cell RNA-seq dataset, the same dataset used in Section 3.7.

We have revised Section 3.8 to explicitly state the data source:"To further explore signaling pathway alterations under the context of metabolic stress, we calculated enrichment scores using the publicly available single-cell RNA-seq data from the GSE145154 dataset, with..."

7) Line 409: "extensive fibrotic lesion" Figure 10A. No fibrotic lesion can be seen in this figure. It would be very difficult to assess fibrosis only with H&E staining. ECM staining should have been done. Also 4 days of HF would not cause much fibrosis formation. Since no specific markers of fibrosis were investigated and the duration of animal model was too short for fibrosis, in this reviewer's opinion the authors are making claims which are not justified by their results. I would strongly advise to remove any claims of fibrosis throughout the manuscript.

Thank you for this critical and insightful comment. We completely agree with your assessment. Without specific fibrosis markers or appropriate staining methods, our claims regarding fibrosis were not justified by the actual experimental evidence. We sincerely apologize for this overstatement and have now carefully reviewed the entire manuscript to remove all claims related to fibrosis.

8) Lines 412-415: The WB is showing that DAPA prevents HF-induced increase in STAT1 expression. It is not showing that DAPA reduced STAT1 activation. A change in protein expression does not necessarily mean a change in activity of that protein. Please rephrase.

We thank the reviewer for this important distinction between STAT1 expression and activation. The reviewer is absolutely correct that our Western blot data measure total STAT1 protein expression levels, not STAT1 phosphorylation status or transcriptional activity. We apologize for the imprecise terminology throughout the manuscript.

We have systematically revised all instances where we incorrectly stated "STAT1 activation" or "STAT1 activity" to accurately reflect that we measured "STAT1 expression" or "STAT1 protein levels."

Besides, we have added this limitation to the Discussion section：“Second, our Western blot data measured total STAT1 protein expression rather than phosphorylation status (p-STAT1 at Tyr701/Ser727) or transcriptional activity. Future studies should include phospho-STAT1 Western blotting, nuclear/cytoplasmic fractionation, STAT1 target gene expression analysis, and ChIP assays to directly assess STAT1 expression. Third, our findings do not establish STAT1 as a direct pharmacological target of DAPA; STAT1 reduction could be an indirect consequence of DAPA's effects on metabolic normalization or oxidative stress reduction. STAT1 loss-of-function studies and drug-target engagement assays would be valuable to establish causality.”

9) Line 431: The authors claim that STAT1 emerged as a key target for DAPA. They have no results to justify this conclusion. Changes in STAT1 expression by DAPA does not mean that STAT1 is a key target for DAPA.

We thank the reviewer for this critical comment. Our wording "key target" overstates the strength of our evidence. We have revised our language to more accurately reflect what our data support.

Besides, we have clarified the discussion: “These findings suggest STAT1 expression may be associated with DAPA's cardioprotective effects, though whether STAT1 is a direct pharmacological target or an indirect consequence of DAPA treatment requires further investigation…Second, our Western blot data measured total STAT1 protein expression rather than phosphorylation status (p-STAT1 at Tyr701/Ser727) or transcriptional activity. Future studies should include phospho-STAT1 Western blotting, nuclear/cytoplasmic fractionation, STAT1 target gene expression analysis, and ChIP assays to directly assess STAT1 expression. Third, our findings do not establish STAT1 as a direct pharmacological target of DAPA; STAT1 reduction could be an indirect consequence of DAPA's effects on metabolic normalization or oxidative stress reduction. STAT1 loss-of-function studies and drug-target engagement assays would be valuable to establish causality.”

10) Lines 438-439: "These in vitro findings reinforce the in vivo results, demonstrating that DAPA protects cardiomyocytes by inhibiting STAT1 activation". Again the authors do not have any data on STAT1 activity. Expression is not activity.

We thank the reviewer for this important distinction between STAT1 expression and activation. The reviewer is absolutely correct that our Western blot data measure total STAT1 protein expression levels, not STAT1 phosphorylation status or transcriptional activity. We apologize for the imprecise terminology throughout the manuscript.

We have systematically revised all instances where we incorrectly stated "STAT1 activation" or "STAT1 activity" to accurately reflect that we measured "STAT1 expression" or "STAT1 protein levels."

Besides, we have added this limitation to the Discussion section：“Second, our Western blot data measured total STAT1 protein expression rather than phosphorylation status (p-STAT1 at Tyr701/Ser727) or transcriptional activity. Future studies should include phospho-STAT1 Western blotting, nuclear/cytoplasmic fractionation, STAT1 target gene expression analysis, and ChIP assays to directly assess STAT1 expression. Third, our findings do not establish STAT1 as a direct pharmacological target of DAPA; STAT1 reduction could be an indirect consequence of DAPA's effects on metabolic normalization or oxidative stress reduction. STAT1 loss-of-function studies and drug-target engagement assays would be valuable to establish causality.”

11) There are several lines in the discussion making claims about activity. The discission needs to be rewritten accordingly.

We sincerely thank the reviewer for this critical observation. The reviewer is absolutely correct that we made imprecise statements regarding STAT1 "activity" and "activation". We have now systematically revised the entire manuscript.
